# Does audiovisual information affect anxiety and perceived pain levels in miniscrew application? — a within-person randomized controlled trial

**DOI:** 10.1186/s40510-019-0281-1

**Published:** 2019-08-01

**Authors:** Berra Calik Koseler, Hilal Yilanci, Sabri Ilhan Ramoglu

**Affiliations:** 1 Private Practice, Istanbul, Turkey; 2 Department of Orthodontics, Faculty of Dentistry, Medipol University, Bağcılar, 34214 Istanbul, Turkey; 3 Department of Orthodontics, Faculty of Dentistry, Altınbaş University, Istanbul, Turkey

**Keywords:** Anxiety, Miniscrew, Pain

## Abstract

**Background:**

Anxiety can cause difficulties during surgical procedures. The main objective of this study was to evaluate changes in patients’ anxiety and perceived pain levels after receiving audiovisual and verbal information about miniscrew application.

**Materials and methods:**

Eighty-eight patients (30 males and 58 females) with a mean age of 18.18 ± 5.39 years who had fixed orthodontic treatment and required miniscrew anchorage took part in this questionnaire-based randomized controlled trial. The participants were randomly allocated to two groups and either watched a video depicting miniscrew application (study group, 44 patients) or were informed verbally about the procedure (control group, 44 patients) before miniscrew placement. The audiovisual information was given via a video containing footage of local anesthesia injection, topical antiseptic application, and miniscrew insertion. The Spielberger State-Trait Anxiety Inventory (STAI) was used to measure anxiety immediately before miniscrew application. Self-drilling miniscrews (8 mm length, 1.5 mm diameter; Aarhus System Miniscrews, American Orthodontics, Washington, USA) were placed in posterior buccal interdental region. Each patient received only one miniscrew. Postoperative pain (PP) was determined using a 100-mm horizontal visual analog scale (VAS).

**Results:**

State and total anxiety scores were significantly higher in the study group than in the control group (*p* = 0.009 and *p* = 0.011 respectively). The mean PP scores (SD) for control and study groups were 12.86 (14.22) and 12.8 (16.22), respectively. The results of Mann–Whitney *U* test showed no significant difference (*p* > 0.05). Participants’ PP scores did not have a significant effect on state, trait, or total anxiety scores. There was a weak but significant positive correlation between trait anxiety and state anxiety scores in both groups.

**Conclusion:**

Using an audiovisual method to inform patients about miniscrew placement increased anxiety levels but did not affect pain perception.

## Introduction

One of the main problems faced by both clinicians and patients in dental procedures is anxiety [1]. It is a complex emotion affected by many factors such as gender, age, socioeconomic status, negative dental experiences, and parental dental anxiety, to name a few [2–4].

Anxious patients are less cooperative during dental procedures, more prone to delay or cancel their appointments, and are usually unsatisfied with dental treatment [5, 6]. In addition, dental care of these patients is challenging for dentists because they may become irritated and frustrated during procedures. As a result, clinicians may also become stressed and take longer to perform operations [7, 8].

There are several self-assessment questionnaires for dental anxiety; however, some of these focus on general dental procedures [9, 10]. The Spielberger State-Trait Anxiety Inventory (STAI) was created to measure anxiety in adolescents and is applicable for use in an orthodontic study because it poses no specific scenarios [11]. It is a self-reported questionnaire comprising two scales for measuring state anxiety and trait anxiety. State anxiety refers to how a person feels in times of fear or danger and is temporary. Trait anxiety refers to a personality characteristic and is stable throughout a person’s life. Total anxiety is determined as the sum of state and trait anxiety scores [12].

Previous research reported that state anxiety levels in patients awaiting orthodontic treatment are high, but normalize within the first year [13]. Yildirim et al. [14] stated that patients’ dental anxiety and state anxiety scores were high before orthodontic treatment, but decreased after 3 months as patients became familiar with their orthodontists and orthodontic treatments. In contrast, a previous study reported that patients who had been treated with extra-oral orthodontic appliances for one year had higher state anxiety levels [15].

For biomechanical reasons, miniscrew anchorage is an essential element of a treatment plan, but surgical procedures such as orthognathic surgery or miniscrew application are associated with pain expectancy and are cited as a major source of anxiety [16, 17]. Patients’ knowledge about the importance of temporary anchorage devices may help to decrease pain expectancy and anxiety.

Providing information before a medical procedure allows patients to understand what to expect, and this may reduce their anxiety levels and stress [18]. Information can be given verbally (written or oral), audiovisually, or through combinations of these modalities [11, 19]. A previous study reported that adolescent orthodontic patients believed that information provided in a multimedia format might be more helpful to reduce their anxiety because they could predict what would actually happen [20]. Wright et al. [21] suggested that verbal information combined with detailed written information during early orthodontic treatment had favorable effects on patients’ compliance but did not affect patients’ treatment-related anxiety levels. However, Srai et al. [11] showed that giving combined multimedia and verbal information or verbal information alone regarding the bonding procedure did not reduce anxiety levels significantly.

The aim of this study was to evaluate the effects of providing verbal or audiovisual information on patients’ anxiety levels before miniscrew application. The null hypothesis was that there would be no difference in anxiety and pain levels when either of the methods is used.

## Materials and methods

Ethical approval was obtained from the Clinical Research Ethics Committee of Bezmialem University in Istanbul, Turkey (No. 2017/12). This prospective and randomized controlled trial evaluated anxiety levels of patients who were given verbal or multimedia-based information before miniscrew placement. This questionnaire-based longitudinal study was carried out at the Bezmialem Vakif University Dentistry Faculty Orthodontic Department.

The study included 88 patients (30 males and 58 females) with a mean age of 18.18 ± 5.39 years. They were recruited from among patients who were in the active phase of fixed orthodontic treatment and were prescribed miniscrew applications based on the need for additional anchorage. Exclusion criteria were (i) prior miniscrew placement, (ii) requiring more than one miniscrew insertion, (iii) requiring assistance to read Turkish, and (iv) refusal to participate in the study. Participants were randomly allocated into either the control (*n* = 44) or study group (n = 44) based on their date of miniscrew procedure. Participants in the control group received verbal information, whereas those in the study group received multimedia information in the form of a video of a patient undergoing miniscrew application. The participants and orthodontists who performed the miniscrew insertions were blinded to group assignments.

All procedures were performed by orthodontists under infiltrative local anesthesia with slightly less than one quarter of the anesthetic cartridge. The sterile implant was carried with a screwdriver. Self-drilling miniscrews (8 mm length, 1.5 mm diameter; Aarhus System Miniscrews, American Orthodontics Washington, USA) were placed in posterior buccal interdental region according to recommended guidelines with no incision or soft tissue removal from the attached gingival prior to insertion. Patients were instructed to maintain good oral hygiene, and no analgesic or antibiotics were prescribed postoperatively.

The audiovisual information was provided with a video that was recorded at the Bezmialem Vakif University Orthodontic Clinic. The video was 1 min and 42 s in length and contained no subtitles or written information. The video showed the procedures of local anesthesia injection, antisepsis with topical application, and miniscrew insertion.

The script of the verbal information was as follows:

First, local anesthetic will be injected. After the injection, an antiseptic solution will be applied. A miniscrew 8 mm in length and 1.5 mm in diameter will be inserted with a screwdriver.

Patients were provided audiovisual or verbal information by the same researcher (BCK) before miniscrew application and patient anxiety levels were determined using the Spielberger STAI just prior to the procedure [12]. The Turkish version of the questionnaire, which was validated and adapted to the Turkish population by Oner and LeCompte [22], was used. The state anxiety scale (STAI-S) contains 20 statements asking how patients feel at that moment, with respondents rating anxiety from one (“not at all”) to four (“very much so”). The trait anxiety scale (STAI-T) also poses 20 questions to which respondents rate anxiety from one (“almost never”) to four (“almost always”) to assess how the subject usually feels, and scores for each subscale range between 20 and 80. The researcher informed patients how to fill out the questionnaire, and written instructions were also added to each questionnaire.

After miniscrew placement, postoperative pain (PP) was assessed with a 100-mm horizontal visual analog scale (VAS). The scale ranged from 0 to 10, with 0 meaning “no pain at all” and 10 meaning “the worst imaginable pain.” Patients were instructed to draw a line perpendicular to the VAS line at the point that expressed their pain severity after miniscrew insertion.

### Statistical analysis

Statistical analysis was performed using IBM SPSS Statistics v.23 software (IBM SPSS, Chicago). The normality of anxiety score results was evaluated using Shapiro–Wilk test. Age and gender of the patients were analyzed with chi-square and Fisher’s exact test. Student’s *t* test was used to compare median state, trait, and total anxiety scores between the study and control groups. Pearson correlation analysis was used to determine the linear relationship between anxiety and pain, and a *p* value < 0.05 was considered significant. Power analysis was performed to determine the minimum number of samples in each group. The necessary sample size to be included in each group was 41 (power: 0.80, α: 0.05). We decided that 44 patients in each group would be suitable for the purposes of this study.

## Results

The participants were recruited between October 2014 and September 2017, and 95 patients filled out the questionnaire. Seven patients were excluded from the analysis because they did not completely fill out the questionnaire. Therefore, 88 patients (30 males and 58 females; mean age 18.18 ± 5.39) were included in the study.

Demographic information about the patients is shown in Table 1. The mean age (SD) of control and study groups were 17.84 (4.91) and 18.52 (5.86), respectively. There was no significant difference in mean age and sex distribution between groups (*p* > 0.05).

**Table 1:** Comparison of patients’ demographic data

	Control group (*n*:44)	Study group (*n*:44)	*z*	*p* ^a^
Age [mean (SD)]	17.84 (4.91)	18.52 (5.86)	960.5	0.950
Gender *n* (%)			*x* ^2^	*p* ^b^
Male	13 (29.5%)	17 (38.6%)	0.809	0.500
Female	31 (70.5%)	27 (61.4%)		

*SD* standard deviation, *n* number of samples

^a^Mann–Whitney *U* test

^b^Fisher’s exact test

The mean state, trait, and total anxiety scores of the control and study groups are shown in Table 2. There was no significant difference in trait anxiety scores between the groups. In the study group, state and total anxiety scores were significantly higher than in the control group (*p* = 0.009 and *p* = 0.011, respectively).

**Table 2:** Evaluation of anxiety scores between the groups

	Control group	Study group	*t*	*p* ^a^
	Mean (SD)	Mean (SD)		
State anxiety	40.36 (9.98)	46.39 (11.09)	2.678	0.009*
Trait anxiety	38.18 (8.14)	40.68 (7.3)	1.516	0.133
Total anxiety	78.55 (15.39)	87.07 (15.4)	2.596	0.011*

*Significant at *p* < 0.05, *SD* standard deviation

^a^Student’s *t* test

The mean PP scores (SD) for control and study groups were 12.86 (14.22) and 12.8 (16.22), respectively. The results of Mann–Whitney *U* test showed no significant difference (*p* > 0.05). Therefore, the null hypothesis was partially rejected (Table 3).

**Table 3:** Evaluation of postoperative pain scores between groups

	*n*	Minimum	Maximum	Mean (SD)	95% Cl	*t*	*p* ^a^
Control group	44	0	50	12.86 (14.22)	8.5–17.2	− 0.218	0.827
Study group	44	0	66	12.8 (16.22)	7.8–17.7		

*SD* standard deviation, *n* number of samples

^a^Mann–Whitney *U* test

The mean PP score (SD) for males and females in the control group were 14.08 (16.06) and 12.35 (13.62), respectively. The results of the Mann–Whitney test revealed no statistically significant difference between female and male patients in the control (*p* = 0.389) or study group (*p* = 0.736). There was also no statistically significant difference in anxiety scores between female and male patients in either group (Table 4).

**Table 4:** Evaluation of anxiety and postoperative pain scores according to gender

	Control group			Study group		
	Mean (SD)		*p*	Mean (SD)		*p*
	Male	Female		Male	Female	
State anxiety	38.77 (9.54)	41.03 (10.24)	0.369^a^	44.47 (12.36)	47.59 (10.26)	0.499^a^
Trait anxiety	35.54 (6.45)	39.29 (8.61)	0.816^a^	40.35 (6.84)	40.89 (7.69)	0.166^a^
Total anxiety	74.31 (12.95)	80.32 (16.17)	0.45^a^	84.82 (17.52)	88.48 (14.08)	0.241^a^
Postoperative pain	14.08 (16.06)	12.35 (13.62)	0.389^b^	8.47 (9.55)	15.52 (18.94)	0.736^b^

*SD* standard deviation, *PP* postoperative pain

^a^Independent *t* test

^b^Mann–Whitney *U* test

Pearson correlation coefficients between anxiety and PP scores are shown in Table 5. No correlation was found between anxiety and PP scores in either group (*p* > 0.05). State, trait, and total anxiety scores were not associated with participants’ PP.

**Table 5:** Relationship between anxiety and PP scores

PP score	Control group		Study group	
	*r*	*p* ^a^	*r*	*p* ^a^
State anxiety	0.238	0.12	0.064	0.68
Trait anxiety	− 0.024	0.877	0.19	0.217
Total anxiety	0.141	0.36	0.136	0.378

*PP* postoperative pain

^a^Pearson correlation analysis

Trait anxiety was significantly associated with state anxiety in both groups (*p* < 0.05) (Table 6). *r* values for the study and control groups were 0.377 and 0.436, respectively, indicating a weak positive correlation.

**Table 6:** Pearson correlation analysis of trait anxiety and state anxiety

State anxiety	Control group		Study group	
	*r*	*p* ^a^	*r*	*p* ^a^
Trait anxiety	0.436	0.003*	0.377	0.012*

*Significant at *p* < 0.05

^a^Pearson correlation analysis

## Discussion

Despite the fact that miniscrew placement is a simple procedure, patients can feel uncomfortable because the procedure is somewhat invasive. We hypothesized that showing the full procedure to patients in a video would reduce anxiety and perceived pain to a greater degree than verbal information. Therefore, we evaluated state and trait anxiety levels before miniscrew placement and pain after miniscrew placement. According to our results, the null hypothesis is rejected.

The state and total anxiety levels in the study group were significantly higher than those of the control group. This indicates that providing video-based information before miniscrew application increases state anxiety levels. This was an unexpected result, because we reasoned that watching the procedure would be more informative than having the procedure explained verbally and may therefore decrease anxiety levels. However, our findings indicate that it had the opposite effect. In contrast, Srai et al. [11] reported that providing additional audiovisual information regarding the bonding procedure significantly reduced state anxiety levels, but the reductions were not clinically important. The difference between these two studies might be related to the procedures. Anxiety levels may be reduced by watching a noninvasive bonding procedure, while this might not apply for miniscrew application.

In the present study, there was no statistically significant difference in anxiety scores between the female and male patients in either group. Many authors have reported that dental anxiety [23] or state anxiety [24] is more common in women than men. In contrast, other studies have shown no relationship between gender and dental or state/trait anxiety levels [11, 13, 14]. These conflicting findings may be associated with cultural differences among the study populations.

The VAS is a 10-cm horizontal linear scale and has been used in many previous studies [25–27]. It was selected for pain assessment because it is a simple and reliable scale for evaluating dental pain [28]. However, it is not practical to standardize pain because patients might have different pain tolerance levels [29].

PP scores (SD) for study and control groups were 12.8 (16.22) and 12.86 (14.22) respectively, and no significant difference was found between two groups. These scores were also lower than those found in other studies; Prabhat et al. [23] reported that a mean PP score of 27.51 (3.41) for miniscrew insertion. Pithon et al. [30] reported a PP score of 3.03 (2.30) after inserting miniscrews under infiltrative local anesthesia with slightly less than one quarter of the anesthetic cartridge, whereas Parabhat et al. [23] inserted the miniscrews after application of topical anesthesia. Discrepancies in PP scores may result from different anesthetic techniques or the amount of anesthetic used; alternatively, providing information prior to miniscrew application might affect the pain perceived by patients.

High pain expectancy during insertion of a miniscrew might be associated with high levels of anxiety. Anxious people tend to exaggerate pain and fear [31], and the relationship between pain and anxiety has been reported in several clinical studies [32, 33]. Canakci et al. [34] reported that anxious patients were more likely to present high pain responses. In contrast, Vallerand et al. [35] reported that providing information about the postoperative period before the procedure significantly increased pain relief without higher analgesic consumption. Kazancıoğlu et al. [24] demonstrated that providing written information about third molar extraction surgery preoperatively might reduce perceived pain. By contrast, in the present study, no correlation was found between anxiety scores and PP scores in both groups.

In the present study, Pearson correlation analysis revealed a significant association between patients’ underlying trait anxiety and their state anxiety, with *r* values of 0.377 and 0.436 for the study and control groups, respectively. These results suggest that if a patient has high trait anxiety, their state anxiety after being informed by verbal or audiovisual methods is likely to remain high regardless of how they are informed. In contrast, Srai et al. [11] also found a significant relationship between underlying trait anxiety and state anxiety, but determined that only 18.7% of the state anxiety was affected by underlying trait anxiety and group allocation. Also, Nigam et al. [36] found that 24% of children with high levels of general anxiety showed high levels of dental anxiety. Furthermore, 56% of children exhibited an association between high dental anxiety and moderate levels of general anxiety. These conflicting results might be due to the multidimensional and multifactorial nature of anxiety.

The personality and attitude of the healthcare provider also have a considerable impact on patients’ experiences. A limitation of this study was that different (but all experienced) orthodontists performed the miniscrew insertions. In addition, the procedure was carried out under local anesthesia. The injection itself may also cause stress and anxiety; however, in this study, it was accepted as a step of the miniscrew procedure and not evaluated separately. This may be considered a limitation of the study. In further research, videos providing information about surgical procedures may incorporate anti-anxiety features such as relaxing music, light and color effects, and portrayals of patients appearing calm, which may have a favorable effect on the viewer’s mood.

## Conclusion

According to the results of this study, verbal and audiovisual information had similar effects on pain perception. However, the audiovisual method caused more anxiety. The reasons for these results should be investigated to better determine how verbal and audiovisual information affect patients and how they should be presented in order to provide the most comfortable experience possible.

## Abbreviations

PP: Postoperative pain
STAI: Spielberger State-Trait Anxiety Inventory
STAI-S: State anxiety scale
STAI-T: Trait anxiety scale
VAS: Visual Analog Scale
